# Toward a Dimensional Assessment of Externalizing Disorders in Children: Reliability and Validity of a Semi-Structured Parent Interview

**DOI:** 10.3389/fpsyg.2020.01840

**Published:** 2020-07-24

**Authors:** Ann-Kathrin Thöne, Anja Görtz-Dorten, Paula Altenberger, Christina Dose, Nina Geldermann, Christopher Hautmann, Lea Teresa Jendreizik, Anne-Katrin Treier, Elena von Wirth, Tobias Banaschewski, Daniel Brandeis, Sabina Millenet, Sarah Hohmann, Katja Becker, Johanna Ketter, Johannes Hebebrand, Jasmin Wenning, Martin Holtmann, Tanja Legenbauer, Michael Huss, Marcel Romanos, Thomas Jans, Julia Geissler, Luise Poustka, Henrik Uebel-von Sandersleben, Tobias Renner, Ute Dürrwächter, Manfred Döpfner

**Affiliations:** ^1^School of Child and Adolescent Cognitive Behavior Therapy (AKiP), Faculty of Medicine, University Hospital Cologne, University of Cologne, Cologne, Germany; ^2^Department of Child and Adolescent Psychiatry, Psychosomatics, and Psychotherapy, Faculty of Medicine, University Hospital Cologne, University of Cologne, Cologne, Germany; ^3^Department of Child and Adolescent Psychiatry and Psychotherapy, Central Institute of Mental Health, Medical Faculty Mannheim, Heidelberg University, Mannheim, Germany; ^4^Department of Child and Adolescent Psychiatry and Psychotherapy, Psychiatric Hospital, University of Zurich, Zurich, Switzerland; ^5^Zurich Center for Integrative Human Physiology, University of Zurich, Zurich, Switzerland; ^6^Neuroscience Center Zurich, University and ETH Zürich, Zurich, Switzerland; ^7^Department of Child and Adolescent Psychiatry, Psychosomatics and Psychotherapy, Medical Faculty, Philipps-University Marburg, Marburg, Germany; ^8^Center for Mind, Brain and Behavior, University of Marburg and Justus Liebig University Giessen, Marburg, Germany; ^9^Department of Child and Adolescent Psychiatry, Psychosomatics and Psychotherapy, University Hospital Essen, University of Duisburg-Essen, Essen, Germany; ^10^LWL-University Hospital for Child and Adolescent Psychiatry, Ruhr-University Bochum, Hamm, Germany; ^11^Department of Child and Adolescent Psychiatry and Psychotherapy, University Medical Center, Johannes Gutenberg University Mainz, Mainz, Germany; ^12^Center of Mental Health, Department of Child and Adolescent Psychiatry, Psychosomatics and Psychotherapy, University Hospital Würzburg, Würzburg, Germany; ^13^Department of Child and Adolescent Psychiatry and Psychotherapy, University Medical Center Göttingen, Göttingen, Germany; ^14^Department of Child and Adolescent Psychiatry, Psychosomatics and Psychotherapy, Tübingen University Hospital, Tübingen, Germany

**Keywords:** structured interview, ADHD, ODD, externalizing disorders, reliability, intraclass correlation coefficient, validity

## Abstract

**Objective:**

This study assesses the reliability and validity of the DSM-5-based, semi-structured *Clinical Parent Interview for Externalizing Disorders in Children and Adolescents* (ILF-EXTERNAL).

**Method:**

Participant data were drawn from the ongoing ESCAschool intervention study. The ILF-EXTERNAL was evaluated in a clinical sample of 474 children and adolescents (aged 6−12 years, 92 females) with symptoms of attention-deficit/hyperactivity disorder (ADHD). To obtain interrater reliability, the one-way random-effects, absolute agreement models of the intraclass correlation (ICC) for single ICC(1,1) and average measurements ICC(1,3) were computed between the interviewers and two independent raters for 45 randomly selected interviews involving ten interviewers. Overall agreement on DSM-5 diagnoses was assessed using Fleiss’ kappa. Further analyses evaluated internal consistencies, item-total correlations as well as correlations between symptom severity and the degree of functional impairment. Additionally, parents completed the German version of the *Child Behavior Checklist* (CBCL) and two DSM-5-based parent questionnaires for the assessment of ADHD symptoms and symptoms of disruptive behavior disorders (FBB-ADHS; FBB-SSV), which were used to evaluate convergent and divergent validity.

**Results:**

ICC coefficients demonstrated very good to excellent interrater reliability on the item and scale level of the ILF-EXTERNAL [scale level: ICC(1,1) = 0.83−0.95; ICC(1,3) = 0.94−0.98]. Overall kappa agreement on DSM-5 diagnoses was substantial to almost perfect for most disorders (0.38 ≤ κ ≤ 0.94). With some exceptions, internal consistencies (0.60 ≤ α ≤ 0.86) and item-total correlations (0.21 ≤ *r*_it_ ≤ 0.71) were generally satisfactory to good. Furthermore, higher symptom severity was associated with a higher degree of functional impairment. The evaluation of convergent validity revealed positive results regarding clinical judgment and parent ratings (FBB-ADHS; FBB-SSV). Correlations between the ILF-EXTERNAL scales and the CBCL *Externalizing Problems* were moderate to high. Finally, the ILF-EXTERNAL scales were significantly more strongly associated with the CBCL *Externalizing Problems* than with the *Internalizing Problems*, indicating divergent validity.

**Conclusion:**

In clinically referred, school-age children, the ILF-EXTERNAL demonstrates sound psychometric properties. The ILF-EXTERNAL is a promising clinical interview and contributes to high-quality diagnostics of externalizing disorders in children and adolescents.

## Introduction

Structured clinical interviews are considered to be the gold standard for diagnosing mental disorders (Rettew et al., 2009; Hoyer and Knappe, 2012; Nordgaard et al., 2013). Accumulating evidence suggests that structured interviews lead to improved diagnostic accuracy and reliability (Frick et al., 2010; Segal and Williams, 2014; Leffler et al., 2015), which can in turn enhance the quality of treatment decision making (Galanter and Patel, 2005). In clinical research, structured interviews are especially used to screen participants for study inclusion or to evaluate psychotherapeutic outcomes (Hoyer and Knappe, 2012; Segal and Williams, 2014). Besides their use in research, such interviews have increasingly found their way into clinical practice as part of a comprehensive and standardized diagnostic process (Frick et al., 2010; Hoyer and Knappe, 2012; Segal and Williams, 2014). Moreover, clinicians in training can also benefit from these instruments, as they cover diagnostic criteria in a systematic manner (Frick et al., 2010; Segal and Williams, 2014; Leffler et al., 2015).

In terms of their degree of structure, clinical interviews can be classified into highly structured versus semi-structured. While the highly structured interviews require only a minimum of training, they leave little flexibility for the interviewer to explore and rate the patient’s symptomatology. Typically, closed-ended questions form a dichotomous assessment, that is, a clinical symptom is either present or absent (Frick et al., 2010; Segal and Williams, 2014; Leffler et al., 2015). Examples of highly structured clinical interviews for assessing children and adolescents include the *NIMH Diagnostic Interview Schedule for Children Version IV* (NIMH DISC-IV; Shaffer et al., 2000), the *Children’s Interview for Psychiatric Syndromes* which encompasses separate child (ChIPS; Weller et al., 1999b) and parent versions (P-ChIPS; Weller et al., 1999a), and the *Mini-International Neuropsychiatric Interview for Children and Adolescents* (Mini-KID; Sheehan et al., 2010). Most of these structured interviews have yet to be revised and validated for DSM-5 (American Psychiatric Association, 2013). As reviewed by Leffler et al. (2015), the current versions of these interviews show high interrater reliability (IRR) and good validity in community and clinical samples. However, such findings may also be attributable to the high degree of structure, and the inherent limited scope for interviewers to form their own clinical judgment (Leffler et al., 2015).

By comparison, semi-structured interviews allow the interviewer to inquire about symptoms, make informed judgments, and score responses in a more flexible manner (e.g., Likert-type scales). While this scoring format requires more extensive training, it can follow a dimensional approach by taking into account the severity of symptoms (Frick et al., 2010; Döpfner and Petermann, 2012; Leffler et al., 2015). Therefore, different interviewers may form disparate judgments, which can in turn result in lower IRR compared to their highly structured counterparts. One of the most prominent semi-structured clinical interviews is the *Schedule for Affective Disorders and Schizophrenia for School Aged Children* (K-SADS; Kaufman et al., 1997) which mainly aims at an early diagnosis of affective disorders but also includes sections on other common mental disorders. Both the parents and their child can be interviewed at the same time. Different editions of the K-SADS exist and the instrument has been evaluated in a variety of populations, with overall good psychometric evidence. Available diagnostic interrater agreement on DSM-IV or DSM-5 externalizing disorders ranges from moderate to almost perfect agreement for several cross-cultural K-SADS adaptations with generally higher agreement in clinical samples (Kim et al., 2004; Ghanizadeh et al., 2006; Ulloa et al., 2006; de la Peña et al., 2018; Nishiyama et al., 2020) than in the community population (Birmaher et al., 2009; Chen et al., 2017). Kariuki et al. (2018) evaluated the attention-deficit/hyperactivity disorder (ADHD) module of the K-SADS in a large community sample and obtained moderate to substantial intraclass correlation (ICC) coefficients for the subdimensions of ADHD. With regard to convergent validity, small to moderate correlations were found between clinical diagnoses and the broadband parent-rated *Child Behavior Checklist* (CBCL) questionnaire (Kim et al., 2004; Birmaher et al., 2009; Brasil and Bordin, 2010; Chen et al., 2017). Furthermore, correlations between clinical diagnoses and the corresponding scales of the CBCL were generally higher than divergent correlations (Birmaher et al., 2009; Chen et al., 2017). Another semi-structured interview is the *Child and Adolescent Psychiatric Assessment* (CAPA; Angold and Costello, 2000) which covers the full range of common mental disorders. With a duration of up to 120 min, it can be very time-consuming to administer (Leffler et al., 2015). In a test-retest study of the CAPA, ICC coefficients for DSM-III-R symptom scale scores ranged from 0.50 for oppositional defiant disorder (ODD) to 0.98 for substance abuse / dependence in self-reports of clinically referred children and adolescents (Angold and Costello, 1995). Furthermore, the CAPA interview has shown good construct validity in relation to ten formulated criteria (Angold and Costello, 2000).

Overall, semi-structured clinical interviews provide a valuable tool for diagnosing mental disorders in children and adolescents (Nordgaard et al., 2013). However, given the evolving conceptualizations of psychopathology, there is a current need for clinical interviews to meet these changing requirements. One such evolving conceptualization considers whether diagnostic domains are best characterized as discrete categories (such as in the DSM-5) or whether they should follow a dimensional approach (Coghill and Sonuga-Barke, 2012; Döpfner and Petermann, 2012). Consequently, state-of-the-art assessment instruments should have the flexibility to allow both a categorical assessment and follow a dimensional approach which allows for varying degrees of severity and functional impairment. To the best of our knowledge, there is no diagnostic system available which meets all of the following criteria: a DSM-5-based, semi-structured clinical interview for externalizing disorders which follows both a categorical and dimensional approach by assessing symptom severity and functional impairment on a Likert scale and includes parallel parent forms with the exact same diagnostic scales.

To meet these criteria, we developed a comprehensive set of clinical parent and patient interviews *Diagnostic System of Mental Disorders in Children and Adolescents – Interview* (DISYPS-ILF; Görtz-Dorten et al., in press), which are part of the German *Diagnostic System of Mental Disorders in Children and Adolescents based on the ICD-10 and DSM-5* (DISYPS-III; Döpfner and Görtz-Dorten, 2017). Of these interviews, the *Clinical Parent Interview for Externalizing Disorders in Children and Adolescents* (ILF-EXTERNAL) covers diagnostic criteria according to the DSM-5 for the following externalizing disorders: ADHD, with the subtypes “combined type,” “predominantly inattentive type,” and “predominantly hyperactive-impulsive type;” ODD; conduct disorder (CD), with the specifier limited prosocial emotions; and disruptive mood dysregulation disorder (DMDD); for further details, see **Materials and Methods**. Besides this categorical assessment, the ILF-EXTERNAL also allows clinical symptoms to be viewed from a dimensional standpoint. A further distinguishing and novel characteristic of the ILF-EXTERNAL is that both the interview and the rating scales for parents, teachers, and patients correspond to the same diagnostic system DISYPS-III (Döpfner and Görtz-Dorten, 2017) and therefore have the exact same diagnostic scales. This allows a specific comparison of ratings of parents, teachers, and patients with clinical judgments. In addition, we sought to psychometrically evaluate this interview in a clinical sample of children with externalizing problems, as this group of children represents the prospective target group for clinical assessment using the ILF-EXTERNAL.

Currently, the ILF-EXTERNAL is being conducted in the multicenter consortium ESCAlife (ESCAlife: Evidence-Based Stepped Care of ADHD along the Lifespan). The purpose of this consortium is to evaluate adaptive interventions for patients diagnosed with ADHD, including 3−6-year-old preschool children (ESCApreschool; Becker et al., 2020), 6−12-year-old school children (ESCAschool; Döpfner et al., 2017), and 12−17-year-old adolescents (ESCAadol; Geissler et al., 2018).

The overall aim of this study is to present the newly developed clinical parent interview ILF-EXTERNAL and its psychometric properties, including (1) descriptive statistics for all scales, (2) internal consistencies and item-total correlations, (3) IRR on the item and scale level, (4) overall agreement on DSM-5 diagnoses, (5) associations between symptom severity and the degree of functional impairment, and (6) convergent and divergent validity in a clinical sample of school-age children with ADHD symptoms.

## Materials and Methods

### Measures

During the ESCAschool study, the below-mentioned measures were collected at several main assessment points (Döpfner et al., 2017). In the present study, measures at baseline (i.e., before any intervention) were analyzed.

#### Clinical Parent Interview for Externalizing Disorders in Children and Adolescents (ILF-EXTERNAL)

The clinical parent interview ILF-EXTERNAL (Görtz-Dorten et al., in press) is part of the DISYPS-III (Döpfner and Görtz-Dorten, 2017). The ILF-EXTERNAL comprises a set of items, each of which explores a DSM-5 symptom criterion. Following a semi-structured approach, clinicians give their own judgment by rating each item on a 4-point Likert scale ranging from 0 (age-typical / not at all), to 3 (very much), with higher scores indicating higher symptom severity. To aid clinical judgment, a short description of the symptom severity is provided for each score. Also, an example sentence of a child’s behavior representing a rating of 3 is given for each item. Item scores of 2 and higher are interpreted as clinically relevant and considered to fulfill the DSM-5 symptom criteria. The ILF-EXTERNAL consists of 18 items assessing ADHD symptoms which can be aggregated into two scales, *Inattention* (nine items) and *Hyperactivity-Impulsivity* (nine items). Together, these 18 items form the *ADHD Symptoms* scale. Additionally, five items assess functioning and psychological strain associated with ADHD symptoms and form the *ADHD Functional Impairment* scale. Moreover, the ILF-EXTERNAL consists of 36 items assessing oppositional and disruptive symptoms which are aggregated to the following scales: *ODD Symptoms* (eight items) and *CD Symptoms* (15 items), which together form the scale *ODD/CD Symptoms* (23 items). Further items form the scales *Disruptive Mood Dysregulation* (five items, three of which are also part of the *ODD Symptoms* scale) and *Limited Prosocial Emotions* (11 items). In addition, five items assess functioning and psychological strain associated with ODD and CD symptoms and form the *ODD/CD Functional Impairment* scale (see Supplementary Table 1 in the online Supplementary Material for a more detailed description of the items forming each scale). Scale scores are computed by averaging the associated item scores. In the present study, the items assessing aggressive and antisocial symptoms from the age of 11 (B06 to B15) were excluded from further analyses due to an obvious floor effect, resulting in the shortened scales *CD Symptoms − short version* (five items) and *ODD/CD Symptoms − short version* (13 items).

#### Child Behavior Checklist for Ages 6−18 (CBCL/6-18R)

To examine convergent and divergent validity, information from the German CBCL/6-18R was used (Arbeitsgruppe Deutsche Child Behavior Checklist, 1998; Döpfner et al., 2014). Originally developed by the Achenbach group (Achenbach, 1991; Achenbach and Rescorla, 2001), the German CBCL/6-18R is a broadband questionnaire comprising 120 items developed to assess behavioral and emotional problems in children and adolescents. Parents rate their child’s behavior on a 3-point scale (0 = “not true,” 1 = “somewhat or sometimes true,” and 2 = “very true or often true”). The items form eight syndrome scales and three broadband scales (*Externalizing Problems, Internalizing Problems, Total Problems*). The German CBCL/6-18R has demonstrated at least satisfactory internal consistencies for the eight syndrome scales with slightly higher values in a large clinical sample than in a community sample (Döpfner et al., 2014). Exceptions are the scales *Thought Problems* (α < 0.70 in both samples) and *Somatic Complaints* (α = 0.65 in the community sample). Internal consistencies were good for the *Externalizing Problems* and *Internalizing Problems* (α > 0.80) and excellent for the *Total Problems* (α > 0.90) in both samples. In cross-cultural analyses, Rescorla et al. (2007) found that parents’ ratings were similar across 31 societies including Germany, indicating the multicultural robustness of the CBCL. Furthermore, the configural invariance of the 8-syndrome structure of the CBCL was confirmed in large cross-cultural studies including Germany (Ivanova et al., 2007, 2019). In the present study, the raw scale scores of the eight syndrome scales and the *Internalizing Problems* and *Externalizing Problems* were used.

#### Symptom Checklist for Attention-Deficit/Hyperactivity Disorder (FBB-ADHS)

The German *Symptom Checklist for Attention-deficit/hyperactivity Disorder* (FBB-ADHS) is part of the DISYPS-III (Döpfner and Görtz-Dorten, 2017). This questionnaire consists of 27 items which form identical scales to those in the ILF-EXTERNAL and an additional six items assessing the child’s competencies. All items are rated on a 4-point Likert scale ranging from 0 (“not at all”) to 3 (“very much”). Psychometric evaluations support the reliability and validity of the FBB-ADHS (Döpfner et al., 2008; Erhart et al., 2008). The present analyses included the scales *Inattention*, *Hyperactivity-Impulsivity*, *ADHD Symptoms*, and *ADHD Functional Impairment*.

#### Symptom Checklist for Disruptive Behavior Disorders (FBB-SSV)

The German *Symptom Checklist for Disruptive Behavior Disorders* (FBB-SSV) is also part of the DISYPS-III (Döpfner and Görtz-Dorten, 2017). The structure and assessment are the same as outlined for the FBB-ADHS. The FBB-SSV includes 46 items which also form identical scales to those in the ILF-EXTERNAL and an additional 12 items assessing the child’s competencies. Psychometric evaluations of the FBB-SSV revealed positive results regarding reliability and validity (Görtz-Dorten et al., 2014). The scales *ODD Symptoms, CD Symptoms, ODD/CD Symptoms, Disruptive Mood Dysregulation, Limited Prosocial Emotions*, and *ODD/CD Functional Impairment* were used in the present study. For the sake of consistency with the scales *CD Symptoms − short version* and *ODD/CD Symptoms − short version* of the ILF-EXTERNAL, the items assessing aggressive and antisocial symptoms from the age of 11 (B06 to B15) from the FBB-SSV were also excluded from further analyses.

### Participants and Procedure

Data collection was based on the ongoing ESCAschool intervention study (target *N* = 521), which is part of the research consortium ESCAlife and involves nine study centers located in Germany (Cologne, Essen, Göttingen, Hamm, Mainz, Mannheim, Marburg, Tübingen, Würzburg). The ESCAschool study investigates an evidenced-based, individualized, stepwise-intensifying treatment program based on behavioral and pharmacological interventions for children diagnosed with ADHD. For further details on the procedures, including inclusion and exclusion criteria, please refer to Döpfner et al. (2017). In the present study, the ILF-EXTERNAL was evaluated using ESCAschool baseline data from 474 children (age range 6−12 years, *M* = 8.9, *SD* = 1.5, 92 females). The assessment of the ILF-EXTERNAL baseline data is part of a screening to check the participants’ eligibility for the ESCAschool study. The screening was conducted at two successive appointments no longer than 8 weeks apart. During the screening, the ILF-EXTERNAL was administered to the parents and was either video- or audio-recorded. About one third of the children (32.5%) were receiving ADHD medication prior to the study. In these cases, parents were asked to describe their child’s behavior with and without medication, resulting in two ratings for each item. For the present analyses, the children’s symptomatology without medication was analyzed. Besides children diagnosed with ADHD, the present sample also included children who did not meet criteria for an ADHD diagnosis (i.e., so-called screening negatives of the ESCAschool study). These screening negatives (*n* = 32, including 9 females) were characterized by subclinical ADHD symptomatology, which allowed us to capture the full spectrum of ADHD symptoms. Descriptive statistics (*M*, *SD*) for all ILF-EXTERNAL scales considering only the screening negatives are reported in Table 2. As can be seen, although these children did not fulfill inclusion criteria for the ESCAschool treatment study, they nevertheless exhibited symptoms of externalizing behavior problems. Clinical diagnoses of ADHD and comorbid externalizing disorders were based on the outcome of the ILF-EXTERNAL. To assess comorbid symptoms, all clinicians applied a clinical diagnostic checklist (DCL-SCREEN) from the DISYPS-III (Döpfner and Görtz-Dorten, 2017). All parents and children gave their assent and written informed consent, and each participating study site received ethical approval (Döpfner et al., 2017). Participant data are presented in Table 1.

**TABLE 1:** Sample characteristics.

	Subsample for the analysis of interrater reliability (*n* = 45)	Total sample (*N* = 474)
Age: mean *(SD)*	9.2 (1.6)	8.9 (1.5)
Male: *n* (%)	34 (75.6)	382 (80.6)
**Diagnosis *n* (%)**		
No ADHD diagnosis	1 (2.2)	32 (6.8)
ADHD – combined type	28 (62.2)	208 (43.9)
ADHD – predominantly inattentive type	14 (31.1)	184 (38.8)
ADHD – predominantly hyperactive-impulsive type	2 (4.4)	50 (10.5)
**Comorbidities *n* (%)**		*n* = 454−465
Internalizing disorders:		
– Anxiety	2 (4.4)	29 (6.4)
– Depression	2 (4.4)	15 (3.1)
Externalizing disorders:		
– Oppositional defiant disorder	23 (51.1)	166 (36.6)
– Disruptive mood dysregulation disorder	6 (13.3)	40 (8.8)
– Conduct disorder	5 (11.1)	28 (6.2)
Other disorders:		
– Obsessive-compulsive disorder	1 (2.2)	2 (0.4)
– Tic disorder	4 (8.9)	24 (5.2)
– Autism spectrum disorder	0	2 (0.4)
**Medication *n* (%)**		*n* = 462
ADHD medication	17 (37.8)	150 (32.5)
**Parents’ primary language *n* (%)**		*n* = 458
German	42 (93.3)	429 (93.7)
**Highest parents’ graduation *n* (%)**		*n* = 455
Higher-track school	24 (53.3)	261 (57.4)
Vocational school	2 (4.4)	26 (5.7)
Medium-track school	14 (31.1)	123 (27.0)
Lower-track school	4 (8.9)	43 (9.5)

*Clinical diagnoses of ADHD and comorbid externalizing disorders were based on the semi-structured clinical interview ILF-EXTERNAL conducted with the parents. Further comorbid symptoms were assessed using a clinical diagnostic checklist. ADHD, attention-deficit/hyperactivity disorder.*

**TABLE 2:** Scale characteristics, Cronbach’s alpha (α) and range of item-total correlations of the ILF-EXTERNAL.

		**Total sample (*N* = 474)**				**Screening negatives (*n* = 32)**	
**Scale**	***k* (items)**	**α**	**Item-total *r* (range)**	**Mean (*SD*)**	***N***	**Mean *(SD)***	***n***
ADHD symptoms	18	0.84	0.21−0.62	1.80 (0.50)	474	1.09 (0.51)	32
– Inattention	9	0.71	0.29−0.49	1.95 (0.48)	474	1.35 (0.49)	32
– Hyperactivity-Impulsivity	9	0.87	0.50−0.68	1.64 (0.73)	474	0.84 (0.62)	32
ADHD functional impairment	5	0.62	0.24−0.48	1.61 (0.59)	472	1.03 (0.49)	31
ODD/CD symptoms−short version	13	0.84	0.25−0.62	0.85 (0.50)	450	0.55 (0.55)	23
– ODD symptoms	8	0.82	0.41−0.61	1.12 (0.63)	451	0.78 (0.68)	24
– CD symptoms−short version	5	0.60	0.22−0.47	0.40 (0.43)	450	0.22 (0.37)	23
Disruptive mood dysregulation	5	0.83	0.53−0.67	1.08 (0.73)	452	0.73 (0.65)	24
Limited prosocial emotions	11	0.77	0.23−0.58	0.50 (0.42)	444	0.37 (0.30)	22
ODD/CD functional impairment	5	0.86	0.61−0.71	0.93 (0.78)	442	0.49 (0.73)	21

*Screening negatives are participants who have been screened for eligibility of the ESCAschool study and are characterized by subclinical ADHD symptoms. ADHD, attention-deficit/hyperactivity disorder; CD, conduct disorder; *k*, number of items which form a particular scale; ODD, oppositional defiant disorder.*

### Subsample for the Analysis of Interrater Reliability

To obtain IRR, a subsample of 45 interviews of the ILF-EXTERNAL was chosen (for the characteristics of this subsample, see Table 1). More specifically, we empirically determined the required sample size as recommended by published guidelines on IRR studies (Kottner et al., 2011). We selected a method for sample size calculation for the ICC coefficient (Zou, 2012) which estimates the required sample (*N*) to achieve a reliability coefficient (*ρ*) that is not less than a prespecified value (*ρ_0_*) with a prespecified assurance probability. The calculations revealed that a minimum of 42 interviews rated by two additional raters (*k* = 3) is required to ensure that the lower limit of a 95% one-sided confidence limit for *ρ* = 0.80 is no less than *ρ_0_* = 0.65 with 80% assurance probability based on the ICC one-way random-effects model (Zou, 2012). Subsequently, 45 interviews (five interviews from one clinician from each of the nine study sites) were randomly selected using the *select cases* function in SPSS. Inclusion criteria for the interview recordings were as follows: A video- or audio-recording had to be present for both parts of the interview, the recordings needed to have sufficient audio quality, the clinical assessment had to follow the ILF-EXTERNAL, and, if possible, both parts of the interview should be conducted by the same interviewer. If it was not possible to rate an interview recording due to violation of the inclusion criteria, another recording from the same interviewer was randomly selected. For one study site, there were only four recordings available from one interviewer; in this case, we therefore included one recording by another interviewer from the same study site. In short, the subsample to obtain IRR consists of 45 recordings of the ILF-EXTERNAL conducted by ten interviewers from nine study sites. Typically, interviewers conducted the first part of the interview, assessing ADHD symptoms, at the first appointment and the second part, assessing ODD/CD symptoms, at the second appointment. For the ADHD part, 37 (82.2%) interviews were conducted with the mother, three (6.7%) with the father, and five (11.1%) with both parents. Similarly, for the ODD/CD part, 40 (88.9%) interviews were conducted with the mother, three (6.7%) with the father, and two (4.4%) with both parents. Regarding the duration of the interviews, the ADHD part had a mean length of 42 min (*SD* = 19 min, range 15 to 88 min) and the ODD/CD part had a mean length of 35 min (*SD* = 20 min, range 5 to 98 min). Thirty-eight interviews were video-recorded and seven were audio-recorded. Regarding ADHD diagnosis, 28 children were diagnosed with ADHD combined type, 14 children with ADHD inattentive type, two children with ADHD hyperactive-impulsive type and only one child was below the cut-off for any ADHD diagnosis. Hence, this sample does not capture the full spectrum of ADHD symptomatology but rather represents a clinical sample of different ADHD subtypes. Sample characteristics are reported in Table 1.

### Interview Training

All interviewers who were involved in recruiting patients for the ESCAschool study were trained psychologists or educationists with a Master’s degree, PhD candidates, or in training to become a child and adolescent psychotherapist/psychiatrist. During the ESCAschool study, all interviewers received a standardized training on administering and scoring the ILF-EXTERNAL, including watching a practice video. All interviewers were encouraged to consult their supervisor if they experienced any difficulties regarding the assessment with the ILF-EXTERNAL. Furthermore, two independent raters were asked to rate a subsample of 45 recordings of the ILF-EXTERNAL to obtain IRR. Both independent raters were PhD students at the University of Cologne and were completing their training as child and adolescent psychotherapists. In addition to the ESCAschool training on the ILF-EXTERNAL outlined above, both raters participated in a 1-day workshop in which they discussed the administration of the ILF-EXTERNAL, including detailed information on the scoring of each item. Both raters were then asked to independently code three practice videos randomly selected from the ESCAschool study, after which they received elaborate feedback from their supervisor and discussed potential difficulties when rating the recordings. Both raters were instructed not to discuss the interviews with each other during the rating process.

### Statistical Analysis

All statistical analyses were performed using SPSS Version 26 (SPSS Inc, Chicago, IL, United States) if not stated otherwise. A first check of the data revealed no considerable floor or ceiling effects of the ILF-EXTERNAL item frequencies (except for the items that had been excluded previously). If more than 10% of the items forming a particular scale were missing, this scale was not computed for the affected participant due to a possible bias of the results (Bennett, 2001). This listwise exclusion criterion was also applied to the scales of the parent questionnaire data. A summary of the valid cases for each analysis is provided in the respective tables.

Besides descriptive statistics (mean scores, standard deviations) for all ILF-EXTERNAL scales, Cronbach’s alpha was computed, with values of >0.70 indicating acceptable internal consistency (Nunnally, 1978). Moreover, the corrected item-total correlations were calculated, with values of >0.30 considered acceptable (Field, 2018).

The ICC coefficient (Shrout and Fleiss, 1979; McGraw and Wong, 1996) was computed to assess IRR between the interviewers and both independent raters. The ICC is one of the most common metrics when assessing IRR of continuous data (LeBreton and Senter, 2008; Hallgren, 2012; Koo and Li, 2016). It should be noted that different formulas exist, each involving distinct assumptions about their calculations and therefore leading to different interpretations (Koo and Li, 2016). We computed the ICC one-way random-effects, absolute agreement model for single rater/measurements ICC(1,1) as well as for measures based on a mean-rating ICC(1,3) with their 95% confidence intervals (CIs). The ICC one-way model was chosen because the physical distance between study centers prevented the same interviewer from measuring all participants which would otherwise qualify for the two-way measurement models (Koo and Li, 2016). Furthermore, we believe that the single rater/measurements model ICC(1,1) is more appropriate than average measures, given that the clinical outcome of ILF-EXTERNAL should be based on one clinician and not on the average information obtained from multiple clinicians (Koo and Li, 2016). Nevertheless, we also present average measurements ICC(1,*k*) to ensure comparability of our results across studies. We also calculated the IRR for both independent raters for all scales of the ILF-EXTERNAL using the two-way random-effects models for single ICC(2,1) and for average ICC(2,2) measurements (Shrout and Fleiss, 1979; McGraw and Wong, 1996). To interpret ICC coefficients, different benchmarks are commonly cited. Cicchetti (1994) provided the following guidelines for interpreting ICC coefficients: poor ≤ 0.40; fair = 0.41–0.59; good = 0.60–0.74; and excellent ≥ 0.75. However, other authors proposed more stringent guidelines: poor ≤ 0.50; moderate = 0.51–0.75; good = 0.76–0.90; and excellent ≥ 0.91 (Koo and Li, 2016). The results are therefore presented using both 0.75 and 0.91 as interpretations of “excellent” reliability. Additionally, to obtain a further estimate on the degree of agreement, pairwise percent agreement was calculated based on integer scale scores (Wirtz and Caspar, 2002) using MATLAB and Statistics Toolbox Release 2018b. It should be noted that percentages of agreement do not correct for agreements that would be expected by chance and therefore, may overestimate the degree of agreement (Wirtz and Caspar, 2002).

Overall agreement on DSM-5 diagnoses was assessed using Fleiss’ kappa (Fleiss, 1971) which is a statistical measure to assess agreement between multiple raters (i.e., the interviewers and both raters) on categorical variables (i.e., the presence or absence of a disorder). While Fleiss’ kappa is a chance-corrected measure, it is dependent on the base rate of each disorder. Especially when the base rate of a disorder is low (*n* < 10), corresponding kappa values should only be interpreted with caution. The presence or absence of a DSM-5-based disorder was derived from the raw interview item scores by symptom counts. For example, if at least four items from the *ODD Symptoms* scale were scored with 2 or higher, these scores were considered to fulfill the diagnosis of ODD. Following common research practice, other exclusion criteria (such as not making the diagnosis of ODD in the presence of DMDD or such as only specifying limited prosocial emotions in the presence of CD) were ignored (Angold and Costello, 1995; de la Peña et al., 2018). Fleiss’ kappa was calculated between the interviewers and two raters. To interpret kappa values, Landis and Koch (1977) suggested the following benchmarks: slight ≤ 0.20; fair = 0.21–0.40; moderate = 0.41–0.60; substantial = 0.61–0.80; almost perfect agreement ≥ 0.81.

Pearson product-moment correlations were computed between the ILF-EXTERNAL scales *ADHD Symptoms*, *ODD/CD Symptoms − short version* and the corresponding scales *ADHD Functional Impairment*, *ODD/CD Functional Impairment* in order to describe the relationship between symptom severity and the degree of functional impairment. To test for significant differences between pairs of correlations, the *cocor* software package for the R programming language (R 3.6.2) was applied (Diedenhofen and Musch, 2015). More specifically, we compared the magnitude of two dependent correlation coefficients with overlapping variables (i.e., the correlations have one variable in common) based on Steiger (1980) modification of Dunn and Clark (1969) *z*-transformation.

Additionally, Pearson product-moment correlations were computed between all ILF-EXTERNAL scales and the corresponding scales in the parent forms (FBB-ADHS; FBB-SSV) in order to evaluate convergent validity between clinical judgment and parent ratings. Two-sided paired samples *t*-tests were used group comparisons between the average scores of the ILF-EXTERNAL scales and the corresponding scales of the parent forms. This analysis allowed us to investigate whether clinician-rated scale scores on the ILF-EXTERNAL differed significantly from ratings on the corresponding parent-rated scales.

To further assess convergent and divergent validity, Pearson product-moment correlations were computed between the ILF-EXTERNAL scales and the eight syndrome scales as well as the *Externalizing Problems* and *Internalizing Problems* of the CBCL/6-18R. The R *cocor* package and Steiger’s test (Steiger, 1980) were again applied to compare the magnitude of two dependent correlations. In particular, we determined whether the correlations of a particular ILF-EXTERNAL scale (e.g., *Inattention*) with the CBCL/6-18R broadband scales (*Externalizing Problems* and *Internalizing Problems*) differed significantly.

## Results

### Scale Characteristics

Table 2 summarizes the mean scores, standard deviations, internal consistencies (Cronbach’s alpha) and the ranges of the item-total correlations for all ILF-EXTERNAL scales. The lowest mean score was observed for the scale *CD Symptoms − short version* (*M* = 0.40, *SD* = 0.43), and the highest mean score for the scale *Inattention* (*M* = 1.95, *SD* = 0.48). As can be expected, given the clinical sample of children with ADHD symptoms, average scale scores were generally higher on the ADHD scales than on the ODD/CD scales. Cronbach’s alpha coefficients for the ILF-EXTERNAL symptom scales were generally acceptable to good, with the exception of the scale *CD Symptoms − short version* (α = 0.60). The scales comprising *Functional Impairment* showed questionable internal consistency for ADHD (α = 0.62) and very good internal consistency for ODD/CD (α = 0.86). Item-total correlations were generally satisfactory (0.21 ≤ *r*_it_ ≤ 0.71) with some exceptions. The following items demonstrated item-total correlations below *r*_it_ = 0.30 (ADHD items: *A01 Careless, A06 Concentration, F05 Interferes with educational activities*. ODD/CD items: *B03 Cruel to animals, B05 Steals without confrontation, C04c Manipulates*). However, excluding any of these items did not noticeably change the Cronbach’s alpha of the respective scales.

### Interrater Reliability

Table 3 presents the IRR of the ILF-EXTERNAL scales, according to the ICC one-way random-effects, absolute agreement model for single ICC(1,1) and average measures ICC(1,3), their respective 95% confidence intervals, and pairwise percent agreement. Regarding the ILF-EXTERNAL symptom scales, all ICC(1,1) coefficients were greater than 0.75, indicating excellent IRR according to Cicchetti (1994) or, following a more stringent interpretation, good to excellent IRR (Koo and Li, 2016). Furthermore, all ICC(1,3) coefficients of the average measurement model were greater than 0.90, indicating excellent IRR of the ILF-EXTERNAL symptom scales (Cicchetti, 1994; Koo and Li, 2016). Regarding the ILF-EXTERNAL scales assessing functional impairment, both the *ADHD Functional Impairment* scale [ICC(1,1) = 0.89; ICC(1,3) = 0.96] and the *ODD/CD Functional Impairment* scale [ICC(1,1) = 0.92; ICC(1,3) = 0.97] demonstrated ICC values in the upper range, indicating very good to excellent IRR by the single and average measurement model (Cicchetti, 1994; Koo and Li, 2016). In addition, pairwise percent agreement was consistently higher than 80%, indicating high agreement between the interviewers and both raters. For the interested reader, results on the IRR on the item level are reported in the Supplementary Table 1. Furthermore, we calculated the IRR for both independent raters for all scales of the ILF-EXTERNAL using the two-way random-effects models for single ICC(2,1) and for average measurements ICC(2,2) (McGraw and Wong, 1996; Shrout and Fleiss, 1979). The results show that all ICC coefficients were 0.90 or greater using single and average measures, indicating excellent IRR of the ILF-EXTERNAL scales (Cicchetti, 1994; Koo and Li, 2016). The results are summarized in the Supplementary Table 2.

**TABLE 3:** Interrater reliability of the ILF-EXTERNAL scales.

**Scale**	**ICC(1,1)**	**95% CI**	**ICC(1,3)**	**95% CI**	**Pairwise percent agreement**	***n***
ADHD symptoms	0.91	0.87−0.95	0.97	0.95−0.98	88.1	45
– Inattention	0.83	0.74−0.90	0.94	0.89−0.96	85.2	45
– Hyperactivity-Impulsivity	0.95	0.91−0.97	0.98	0.97−0.99	82.2	45
ADHD functional impairment	0.89	0.82−0.94	0.96	0.93−0.98	80.7	39
ODD/CD symptoms−short version	0.94	0.90−0.96	0.98	0.97−0.99	91.1	45
– ODD symptoms	0.94	0.90−0.96	0.98	0.96−0.99	83.7	45
– CD symptoms−short version	0.90	0.85−0.94	0.97	0.94−0.98	88.2	44
Disruptive mood dysregulation	0.90	0.85−0.94	0.97	0.94−0.98	83.7	45
Limited prosocial emotions	0.93	0.89−0.96	0.98	0.96−0.99	86.7	41
ODD/CD functional impairment	0.92	0.86−0.96	0.97	0.95−0.99	85.2	31

*ADHD, attention-deficit/hyperactivity disorder; CD, conduct disorder; CI, confidence interval; ICC, intraclass correlation; ICC(1,1), one-way random-effects, absolute agreement model for single rater/measurements; ICC(1,3), one-way random-effects, absolute agreement model based on a mean-rating; ODD, oppositional defiant disorder.*

### Agreement on DSM-5 Diagnoses

Table 4 presents overall agreement on DSM-5 diagnoses assessed using Fleiss’ kappa values, their corresponding 95% confidence intervals, and pairwise percent agreement. Following the benchmarks of Landis and Koch (1977) diagnostic agreement ranged from fair (ADHD hyperactive-impulsive type: κ = 0.38), through moderate (DMDD: κ = 0.55), substantial (ADHD combined type: κ = 0.71; ADHD inattentive type: κ = 0.71; any ADHD: κ = 0.74) to almost perfect agreement (ODD: κ = 0.82; conduct disorder: κ = 0.94; with its specifier limited prosocial emotions: κ = 0.82). However, due to the low base rate of the diagnoses ADHD hyperactive-impulsive type (*n* = 2) and CD (*n* = 6) in the subsample, agreement on these two disorders should be interpreted with caution. In particular, pairwise percent agreement mainly seems to reflect agreement by chance. With regard to the remaining DSM-5 diagnoses, agreement could be estimated more reliably.

**TABLE 4:** Agreement on DSM-5 diagnoses in the subsample for the analysis of interrater reliability.

**DSM-5 Diagnosis**	**Fleiss’ Kappa**	**95% CI**	**Pairwise percent agreement**	**Base rate *n***
Any ADHD	0.74	0.74−0.75	100	44
ADHD: combined type	0.71	0.70−0.71	86.7	29
ADHD: predominantly inattentive type	0.74	0.74−0.75	89.6	13
ADHD: predominantly hyperactive-impulsive type^a^	0.38	0.37−0.38	95.6	2
Oppositional defiant disorder	0.82	0.82−0.83	91.1	24
Disruptive mood dysregulation disorder	0.55	0.54−0.55	88.2	10
Conduct disorder^a^	0.94	0.94−0.95	98.5	6
– Specifier: Limited prosocial emotions	0.82	0.81−0.82	91.1	18

*The presence of a disorder was derived from the raw interview item scores by symptom counts. ^*a*^Diagnostic agreement should be interpreted with caution due to a low base rate (*n* < 10). Sample size *n* = 45: ADHD, attention-deficit/hyperactivity disorder.*

### Correlations Between ILF-EXTERNAL Symptom Scales and Functional Impairment

Regarding the association between symptom severity and the degree of functional impairment, Pearson correlations revealed a moderate to large (*r* = 0.50) association between the scales *ADHD Symptoms* and *ADHD Functional Impairment*. In turn, there was a strong positive association between the scales *ODD/CD Symptoms − short version* and *ODD/CD Functional Impairment* (*r* = 0.67). Furthermore, the scale *ADHD Symptoms* correlated significantly more strongly with the scale on functional impairment associated with ADHD than with the *ODD/CD Functional Impairment* scale (*z* = 3.92, *p* < 0.001). Likewise, the scale *ODD/CD Symptoms* correlated significantly more strongly with the scale on ODD/CD-related functional impairment than with the *ADHD Functional Impairment* scale (*z* = −5.41, *p* < 0.001).

### Convergent and Divergent Validity

Table 5 compares the ILF-EXTERNAL scales and the corresponding scales of the parent forms (FBB-ADHS; FBB-SSV). Pearson correlations were moderate to high and significant (0.57 ≤ *r* ≤ 0.78, *p* < 0.001), indicating convergent validity between clinical judgment and parent ratings. Overall, ratings on most ILF-EXTERNAL scale scores differed significantly from ratings on the corresponding scales of the parent forms (*p* < 0.05), with the exception of the scales *ADHD Symptoms* (*p* = 0.051) and *CD Symptoms − short version* (*p* = 0.260). Furthermore, mean scale scores on the parent forms were higher than the corresponding clinical judgment (exceptions: *ADHD Symptoms* and *Hyperactivity-Impulsivity*).

**TABLE 5:** Comparisons of the ILF-EXTERNAL scales and the corresponding parent forms (FBB-ADHS and FBB-SSV).

**Scale**	**ILF-EXTERNAL: Mean (SD)**	**FBB (parents): Mean (SD)**	***r***	**Paired samples *t*-test**	***p***	***n***
ADHD Symptoms	1.83 (0.48)	1.79 (0.56)	0.69***	*t*(425) = 1.95	0.051	426
– Inattention	1.98 (0.45)	2.03 (0.57)	0.58***	*t*(420) =−2.35	0.019	421
– Hyperactivity-Impulsivity	1.67 (0.71)	1.59 (0.73)	0.78***	*t*(422) = 3.78	<0.001	423
ADHD Functional Impairment	1.62 (0.57)	1.74 (0.69)	0.59***	*t*(400) =−4.16	<0.001	401
ODD/CD Symptoms−short version	0.84 (0.49)	0.97 (0.54)	0.74***	*t*(411) = −6.84	<0.001	412
– ODD Symptoms	1.12 (0.63)	1.38 (0.70)	0.72***	*t*(404) = −10.21	<0.001	405
– CD Symptoms−short version	0.40 (0.43)	0.42 (0.44)	0.65***	*t*(407) = −1.18	0.260	408
Disruptive Mood Dysregulation	1.06 (0.72)	1.21 (0.72)	0.67***	*t*(411) = −4.72	<0.001	412
Limited Prosocial Emotions	0.49 (0.41)	0.68 (0.54)	0.63***	*t*(406) = −8.96	<0.001	407
ODD/CD Functional Impairment	0.94 (0.78)	1.39 (0.82)	0.57***	*t*(380) = −11.75	<0.001	381

ADHD, attention-deficit/hyperactivity disorder; CD, conduct disorder; ODD, oppositional defiant disorder; ILF-EXTERNAL, Clinical Parent Interview for Diagnosing Externalizing Disorders in Children and Adolescents; FBB, parent-rated symptom checklists for the assessment of ADHD symptoms and symptoms of disruptive behavior disorders; ***p < 0.001.

In addition, Table 6 summarizes Pearson correlations between the ILF-EXTERNAL scales and the eight CBCL/6-18R syndrome scales as well as the CBCL/6-18R broadband scales *Externalizing Problems* and *Internalizing Problems*. Overall, correlations between the ILF-EXTERNAL scales and the CBCL *Externalizing Problems* were moderate to high and significant (0.33 ≤ *r* ≤ 0.69, *p* < 0.001). As can be expected, the highest observed correlations of the CBCL *Externalizing Problems* were with the ILF-EXTERNAL scales *ODD Symptoms*, *CD Symptoms − short version*, and *ODD/CD Symptoms − short version* scales (0.58 ≤ *r* ≤ 0.69). Furthermore, the ILF-EXTERNAL *Inattention* scale was most strongly associated with the CBCL syndrome scale *Attention Problems* (*r* = 0.39). As can be expected, the ILF-EXTERNAL scales were more strongly associated with the CBCL *Externalizing Problems* than the *Internalizing Problems*. When comparing the correlation coefficients of both CBCL problem scales, we found that all ILF-EXTERNAL scales were significantly more strongly associated with the CBCL *Externalizing Problems* (0.001 ≤ *p* ≤ 0.005). Taken together, these results provide support for the convergent and divergent validity of the ILF-EXTERNAL.

**TABLE 6:** Correlations of the ILF-EXTERNAL scales and the Child Behavior Checklist (CBCL/6-18) syndrome scales.

	**CBCL/6-18**											
	**Anxious / Depressed**	**Withdrawn / Depressed**	**Somatic complaints**	**Social problems**	**Thought problems**	**Attention problems**	**Rule-Breaking behavior**	**Aggressive behavior**	**Externalizing problems**	**Internalizing problems**	**Externalizing vs. Internalizing problems**	
		
**Scale**											***z***	***p***
ADHD Symptoms	0.21***	0.02	0.12*	0.37***	0.26***	0.31***	0.38***	0.50***	0.49***	0.17***	6.65	<0.001
– Inattention	0.16***	0.16***	0.14**	0.29***	0.21***	0.39***	0.29***	0.32***	0.33***	0.19***	2.79	0.005
– Hyperactivity-Impulsivity	0.18***	–0.07	0.08	0.31***	0.21***	0.17***	0.32***	0.47***	0.44***	0.10*	6.90	<0.001
ODD/CD Symptoms−short version	0.22***	0.12*	0.15**	0.35***	0.21***	0.21***	0.59***	0.68***	0.69***	0.21***	11.17	<0.001
– ODD Symptoms	0.24***	0.13**	0.15**	0.35***	0.21***	0.21***	0.52***	0.64***	0.64***	0.23***	9.27	<0.001
– CD Symptoms−short version	0.11*	0.06	0.08	0.24***	0.14**	0.16**	0.56***	0.54***	0.58***	0.11*	10.03	<0.001
Disruptive Mood Dysregulation	0.26***	0.15**	0.13**	0.32***	0.21***	0.18***	0.39***	0.59***	0.55***	0.24***	6.69	<0.001
Limited Prosocial Emotions	0.20***	0.21***	0.10*	0.29***	0.21***	0.25***	0.44***	0.43***	0.46***	0.22***	4.99	<0.001

Sample size *n* = 407; ADHD, attention-deficit/hyperactivity disorder; CD, conduct disorder; ODD, oppositional defiant disorder; ILF-EXTERNAL, Clinical Parent Interview for Diagnosing Externalizing Disorders in Children and Adolescents. *p < 0.05; **p < 0.01; and ***p < 0.001.

## Discussion

This study presents the DSM-5-based, semi-structured, clinical parent interview ILF-EXTERNAL and its psychometric properties in a clinical sample of school-age children with ADHD symptoms. The results suggest that the ILF-EXTERNAL is a promising and overall reliable and valid clinical interview for diagnosing externalizing disorders in children and adolescents.

Regarding scale reliability, Cronbach’s alpha coefficients for the ILF-EXTERNAL scales were generally acceptable to good. Accordingly, those items which were aggregated to form a particular scale predominantly seem to measure a common construct. One exception is the *CD Symptoms − short version* scale (α = 0.60). Similar internal consistency of the *CD Symptoms* scale was reported for the DISC version 2.3 (α = 0.59, Frick et al., 2010). We believe that for the following reasons, this rather low internal consistency is unsurprising: First, we excluded the items B06 to B15, assessing aggressive and antisocial symptoms from the age of 11, which resulted in a shortened scale of only five items. Second, with a low mean score (*M* = 0.40; *SD* = 0.43), the scores of the remaining items of this shortened scale displayed a skewed distribution. Third, these symptoms represent a heterogeneous group of symptoms, which may have impaired the reliability of this scale (Frick et al., 2010). Similarly, the *ADHD Functional Impairment* scale demonstrated low internal consistency (α = 0.62), which might also be explained by the heterogeneity of the items. However, the *ODD/CD Functional Impairment* scale showed very good internal consistency (α = 0.86). In addition, item-total correlations were generally satisfactory with some exceptions. Although some items demonstrated item-total correlations below *r*_it_ = 0.30, excluding any of these items did not noticeably change the Cronbach’s alpha of the respective scales.

Having calculated the ICC one-way random-effects model for single ICC(1,1) and average ICC(1,3) measurements, ICC coefficients demonstrated “very good” to “excellent” IRR for all scales (Cicchetti, 1994; Koo and Li, 2016). Most IRR studies on broadband clinical interviews assessing children and adolescents did not provide IRR results on the scale level. One previous study assessed externalizing symptoms in children and adolescents using a modified ADHD-ODD scale of the K-SADS (Jans et al., 2009). This modified scale was based on a dichotomous assessment of the DSM-IV-based ADHD and ODD criteria, leading to a sum score. Pearson correlations revealed a strong positive association (*r* = 0.98) between the sum scores of the interviewers and the sum scores from independent raters. However, it should be noted that ICC might be a more appropriate measure to assess IRR than Pearson correlations. While the Pearson correlation coefficient indicates the strength of the linear relationship between two variables, a high correlation may be observed even though agreement is poor (Bland and Altman, 1986; Gisev et al., 2013). Another study assessed IRR of the ADHD subdimensions in the K-SADS using ICC (Kariuki et al., 2018). The results indicated moderate to good IRR for the inattentive subtype (ICC = 0.76), hyperactive-impulsive subtype (ICC = 0.41), combined type (ICC = 0.77), and any ADHD type (ICC = 0.64). While the authors calculated the one-way random-effects model, it remains unclear whether they relied on single or average measurements, which limits the interpretation of their results. Although our ICC coefficients were consistently higher on all ADHD scales, a comparison with the aforementioned study must be treated with caution for the following reasons: First, the authors validated the ADHD subdomains in a community sample, while our results were based on clinically referred children. Second, the authors only obtained IRR estimates from 20 children, while we empirically calculated our required sample size and based our IRR results on twice as many children. Overall, our study demonstrates high IRR and addresses the aforementioned research gap, providing valuable information regarding the psychometric quality on the scale level. These findings were largely confirmed even on the single-item level (see Supplementary Table 1).

Diagnostic agreement between the interviewers and both independent raters was “substantial” to “almost perfect” for most disorders with the exceptions of ADHD hyperactive-impulsive type and DMDD (Landis and Koch, 1977). With regard to diagnosing ADHD and its subtypes, we found substantial agreement for any ADHD diagnosis, for ADHD combined type, and for ADHD inattentive type. However, these results should be discussed within the scope of the subsample. The composition of this subsample may have influenced agreement estimates, particularly because of the high base rate of ADHD diagnoses (i.e., 44/45 children). Although both independent raters were not aware of this high base rate, the sole fact that almost all children exhibited clinically relevant symptoms (i.e., scorings of 2 or 3 on each item) may have led to an uneven distribution of item scorings and thus, possible overestimation of agreement on ADHD diagnoses. For example, the “perfect” pairwise agreement of 100% for any ADHD diagnosis (κ = 0.74) rather seems to reflect an overestimation of agreement due to sampling issues. Concerning diagnostic agreement on ADHD hyperactive-impulsive type, we found rather low Fleiss’ kappa agreement (κ = 0.38) but almost perfect pairwise agreement (95.6%). Although this finding might seem somewhat perplexing, it can be explained as follows: Considering that Fleiss’ kappa is influenced by the base rate of observations (Wirtz and Caspar, 2002), the agreement on ADHD hyperactive-impulsive type seems to primarily reflect sampling issues due its very low base rate (*n* = 2) in our subsample. This low base rate, in turn, influences pairwise percent agreement which does not correct for agreement that would be expected by chance. For example, even if both raters agreed on *no* ADHD hyperactive-impulsive diagnosis for all 45 participants, they still would have demonstrated agreement in 43/45 cases.

As a newly developed clinical interview with a semi-structured format, it is particularly essential to compare diagnostic interrater agreement of the ILF-EXTERNAL with that from other semi-structured interviews. The degree of agreement on any ADHD diagnosis was comparable with other findings in clinical samples using the K-SADS (0.42 ≤ κ ≤ 0.92; Kim et al., 2004; Ghanizadeh et al., 2006; Ulloa et al., 2006; de la Peña et al., 2018; Nishiyama et al., 2020). Furthermore, our results regarding diagnostic agreement on ADHD subtypes were also relatable to previous literature. Having calculated kappa agreement using the MINI-KID interview in a clinical sample, Sheehan et al. (2010) reported almost perfect agreement for ADHD combined type (κ = 0.90) and ADHD inattentive type (κ = 0.93) and substantial agreement for ADHD hyperactive-impulsive type (κ = 0.65). Interestingly, high diagnostic agreement on diagnosing ADHD combined type (κ = 0.86) and ADHD inattentive type (κ = 0.78) was also reported in a clinical sample of children with ADHD symptoms (Power et al., 2004).

With regard to comorbid externalizing disorders, the degree of diagnostic agreement was comparable with other findings in clinical samples using the K-SADS for ODD (0.69 ≤ κ ≤ 0.80; Ghanizadeh et al., 2006; de la Peña et al., 2018) DMDD (κ = 0.53; de la Peña et al., 2018), and CD (0.78 ≤ κ ≤ 1.0; Ghanizadeh et al., 2006; Ulloa et al., 2006; de la Peña et al., 2018). Although our results concerning CD should be interpreted with caution due to its low base rate in the subsample (*n* = 6), these results were also in line with previous studies reporting the highest agreement on this diagnosis (Ghanizadeh et al., 2006; Ulloa et al., 2006). We suggest that this finding may be attributable to the clinical presentation of CD symptoms, which are clear to observe and unambiguous to score. Agreement on the specifier limited prosocial emotions was classified if symptoms in at least two out of four categories were considered as clinically relevant. While previous research observed fair agreement (κ = 0.29; de la Peña et al., 2018) we found very high diagnostic agreement on this specifier (κ = 0.82), which again, may be attributable to our sample characteristics.

The ranges of diagnostic agreement reported in the literature might arise from differences in the administration of the interview (e.g., parents or children as primary informant), the respective study samples (e.g., children or adolescents), methodological issues (e.g., number of raters or amount of training received on administering the interview), or the sample population (community vs. clinical) and its characteristics (e.g., base rates of disorders). Notably, diagnostic agreement is often higher in clinical than in community samples (Chen et al., 2017). One basic criticism of clinical samples is that they typically only include patients with clear and severe symptoms. Consequently, the patients’ symptoms can be easily recognized and scored, which may lead to overestimated reliability results, an effect which is also referred to as spectrum bias (Ranshoff and Feinstein, 1978).

Overall, while these reliability results and their corresponding coefficients yield important empirical findings, these labels do not indicate their practical or clinical relevance (Kottner et al., 2011). In other words, even though we obtained very good to excellent IRR and diagnostic agreement results, discrepancies between ratings nevertheless occurred, which warrant further discussion. We critically explored discrepancies between the interviewers and both raters and propose the following reasons for rater disagreement: (1) In terms of the administration of the ILF-EXTERNAL, we noted that some interviewers explored the frequency and intensity of each symptom more thoroughly than did others. This possible lack of clinical information may have affected the scorings of both independent raters. Moreover, (2) noise disturbances during the recordings may have affected the raters, and (3) information variance (i.e., the interviewers may have integrated information prior to the interview into their ratings, as well as 4) interpretation variance (i.e., different raters may have subjective ideas about weighting of symptoms) might have arisen (Hoyer and Knappe, 2012).

A further finding was that higher symptom severity was associated with a higher degree of functional impairment. This result highlights the importance of the current DSM practice of considering a clinical significance criterion (Spitzer and Wakefield, 1999) which requires symptoms to be associated with clinically significant psychological strain and functional impairment in social, occupational, or other areas of life to warrant a diagnosis (American Psychiatric Association, 2013). Results from a large meta-analysis confirmed the relationship between ADHD subtypes and multiple domains of functional impairment (Willcutt et al., 2012).

Regarding convergent and divergent validity, we found moderate to strong correlations between the ILF-EXTERNAL scales and the scales of the German CBCL/6-18R covering similar symptoms. Furthermore, the ILF-EXTERNAL scales were significantly more strongly associated with the CBCL *Externalizing Problems* than with the *Internalizing Problems*, indicating construct validity. These results are largely consistent with previous studies reporting small to moderate relations between the CBCL and clinical diagnoses from semi-structured interviews for the assessment of clinical symptoms in children and adolescents (Kim et al., 2004; Birmaher et al., 2009; Brasil and Bordin, 2010; Chen et al., 2017). Moreover, correlations of the ILF-EXTERNAL scales with the corresponding CBCL scales were generally higher than correlations with the non-corresponding CBCL scales. Similar findings have also been reported in the community population (Kim et al., 2004; Birmaher et al., 2009; Chen et al., 2017). However, limitations of these findings are that they often rely solely on broad diagnostic categories such as “ADHD” without specification of its subtypes (Birmaher et al., 2009; Chen et al., 2017), “any disruptive disorder” (Brasil and Bordin, 2010), or that their results are based on small (i.e., less than *N* = 100) sample sizes (Kim et al., 2004; Brasil and Bordin, 2010). We therefore extended these findings by reporting validity results on diagnostic scales in a larger sample. A further strength of our study is that we included parent forms (FBB-ADHS; FBB-SSV) which cover the same DSM-5 symptoms as the ILF-EXTERNAL. This distinguishing and novel characteristic allowed us to specifically compare ratings between parental and clinical judgments. While our results indicate moderate to substantial convergence between parent ratings and clinical judgments, we believe that this convergence is not sufficiently strong to argue that raters could be seen as interchangeable. In contrast, Boyle et al. (2017) challenged that structured clinical interviews may be replaced by self-completed problem checklists as a time- and cost-effective alternative. One basic criticism was that *“the dependence on respondents in these interviews is similar to the dependence on respondents completing a checklist on their own except for the potential error introduced by interviewer characteristics and interviewer–respondent exchanges”* (Boyle et al., 2017, p. 2). While we agree with this view inasmuch as clinical interviews should provide additional value to questionnaire data such as problem checklists, close inspection of our results revealed the following: Although we found moderate to large correlations between clinician and parent ratings, comparisons of the absolute scale scores revealed significant differences between the ratings on several scales. This indicates that both perspectives are complementary and that both are necessary for an informed clinical diagnosis. On top of that, similar recommendations are made by the German interdisciplinary evidence- and consensus-based (S3) guidelines on the clinical assessment of ADHD (Association of Scientific Medical Societies in Germany AWMF, 2018).

In terms of limitations, one drawback of the present study is that parents were the only informants for both the interview and the questionnaires. Hence, no information was available from the children themselves. However, we believe that this limitation is surmountable given that parents are typically better informants regarding their children’s externalizing behavior problems than their children.

Another significant aspect to consider is the composition of the subsample for the analysis of IRR. We concede that the high base rate of ADHD diagnoses may have influenced interrater agreement. As percentages agreement do not correct for agreements that would be expected by chance, they may overestimate the degree of agreement. In particular, the almost perfect percentages of agreement on some diagnoses rather seem to reflect an overestimation due to chance agreement and sampling issues.

While agreement between parent and teacher ratings on childhood diagnoses is typically quite low (Willcutt et al., 2012) studies investigating interrater agreement between interviewers using clinical interviews yield higher estimates. We concede that these higher agreement estimates may be explained as follows: (1) Intensive rater trainings on the administration and scoring of a clinical interview may lead to more homogenous ratings, and thus, higher rates of agreement. (2) Within research settings, it is common practice to classify agreement on diagnoses based on raw interview item scores by symptom counts. However, this approach may overestimate diagnostic agreement because additional criteria for an informed clinical diagnosis are not further considered. (3) As the interviews are video- or audio-recorded, the interviewers and raters have the exact same informants (e.g., parents) with the exact same information. This approach results in higher agreement estimates compared to other forms of reliability, e.g., test-retest reliability where the same informant is interviewed twice but may provide different information (Angold and Costello, 1995).

Finally, the factor structure of the ILF-EXTERNAL has not yet been validated. While this clinical interview comprises a set of items with each item exploring a DSM-5 symptom criterion, it remains unclear whether this DSM-5-based factor structure can be replicated empirically. For this reason, a follow-up study exploring the factor structure of the ILF-EXTERNAL using correlated factor models and bifactor models is planned. Nevertheless, it should be noted that the factor structure of the corresponding DISYPS parent forms, FBB-ADHS and FBB-SSV, has been confirmed (Erhart et al., 2008; Görtz-Dorten et al., 2014).

We suggest that future studies evaluating psychometric properties of structured clinical interviews should include ratings of symptom severity on the scale level as part of a dimensional approach. Ideally, specific aspects covering functioning and psychological strain could also be included.

## Conclusion

The aim of this study was to assess the reliability and validity of a DSM-5-based, semi-structured parent interview for diagnosing externalizing disorders in children and adolescents. In clinically referred, school-age children, the ILF-EXTERNAL demonstrates sound psychometric properties in terms of IRR on the item and on the scale level, rater agreement on most DSM-5 diagnoses, internal consistency, and convergent and divergent validity. In line with current literature and the DSM practice to consider functional impairment as prerequisite for making a diagnosis, higher symptom severity was associated with a higher degree of functional impairment. Having developed a comprehensive set of clinical parent and patient interviews (DISYPS-ILF), we hope to contribute to a high-quality standard of diagnosing mental disorders in children and adolescents.

## Data Availability Statement

The raw data supporting the conclusions of this article will be made available by the authors, without undue reservation.

## Ethics Statement

The studies involving human participants were reviewed and approved by Ethical approval has been obtained for the study centre Cologne by the University of Cologne (ID 15–216), for the study centre Essen by the University Duisburg-Essen (ID 17–7404-BO), for the study centre Hamm by the Ruhr-University Bochum (ID 15–5564), for the study centre Göttingen by the University Medical Center Göttingen (ID 3/3/17), for the study centre Mainz by the Federal Medical Association of Rhineland-Palatinate (ID 837.237.17 [11071]), for the study centre Mannheim by the Ruprecht-Karls- University Heidelberg (ID 2015-646 N-MA), for the study centre Marburg by the Philipps-University Marburg (ID Studie 03/16), for the study centre Tübingen by the Eberhard-Karls-University Tübingen (ID 791/2015BO2), and for the study centre Würzburg by the Julius-Maximilians-University Würzburg (ID 332/15_z). Written informed consent to participate in this study was provided by the participants legal guardian/next of kin.

## Author Contributions

A-KTh developed the first draft of the manuscript, rated the data (Rater 1), analyzed the data, and was involved in the recruitment and data acquisition of the ESCAschool study site in Cologne and was co-author of the DISYPS-ILF. MD was principal investigator of the ESCAschool trial, developed the basic ESCAschool study design, was part of the ESCAlife-Consortium, was head of the Cologne recruiting center for the ESCA children trials (ESCApreschool, ESCAschool, and ESCAadol), developed the DISYPS-III system, and the DISYPS-ILF, and critically revised the manuscript. AG-D developed the DISYPS-III system and the DISYPS-ILF, was involved in rater training of the ESCAschool study, and critically revised the manuscript. PA was a scientific staff member at the Cologne study site, was involved in patient recruitment and data acquisition, and critically revised the manuscript. CD heads the telephone-assisted self-help trial performed in one treatment arm of the ESCAschool study and critically revised the manuscript. NG rated the data (Rater 2) and critically revised the manuscript. CH was involved in the development of the ESCAschool research proposal and organization of the study, and critically revised the manuscript. LJ and A-KTr coordinated ESCAschool, contributed to the management and organization of the study, and critically revised the manuscript. EW coordinated the Cologne study site, contributed to the implementation of the study, provided supervision for patient treatment, and critically revised the manuscript. TB coordinated the ESCAlife consortium and was the co-principal investigator in the ESCAschool study, and made substantial contributions to the conception and design of the ESCAschool research proposal, heads the Mannheim study site of ESCAschool, and critically revised the manuscript. DB made substantial contributions to the conception and design of the ESCAschool research proposal and the neuropsychological research battery, heads the sub-project ESCAbrain, and critically revised the manuscript. SM heads the Mannheim study site of ESCAschool, coordinates the ESCAlife consortium, made substantial contributions to the conception of the neuropsychological research battery and the neurofeedback protocol, and critically revised the manuscript. SH coordinated the ESCAlife consortium, made substantial contributions to the conception of the neuropsychological research battery and the neurofeedback protocol, and critically revised the manuscript. KB heads the Marburg study site of ESCAschool, was PI for the ESCApreschool study, is a member of the ESCAlife consortium, was a co-applicant of the ESCAlife research project, made substantial contributions to the conception and design of the ESCAlife study, and critically revised the manuscript. JK coordinated ESCAschool at the Marburg study site, contributed to the management and organization of the study, was involved in patient recruitment and data acquisition, and critically revised the manuscript. JH heads the Essen study site of ESCAschool, made substantial contributions to the conception and design of the ESCAschool study, and critically revised the manuscript. JW coordinated ESCAschool at the Essen study site, contributed to the management and organization of the study, and critically revised the manuscript. MHo heads the Hamm/Bochum study site of ESCAschool, was involved in the development of the ESCAschool research proposal, and critically revised the manuscript. TL heads the research department of the Hamm/Bochum study site of ESCAschool, contributed to the implementation of the study in Hamm, supervised the realization of the trial in Hamm, and critically revised the manuscript. MHu heads the Mainz study site of ESCAschool, was involved in the implementation of the ESCAlife projects, and critically revised the manuscript. MR was involved in the planning of the ESCAlife research projects and application for funding, was co-PI for the ESCAadol trial, was involved in the implementation of the ESCAlife projects at the Würzburg study site, and critically revised the manuscript. TJ was involved in the planning of the ESCAlife research projects and application for funding, was co-PI for the ESCAadol trial and was involved in the implementation of the ESCAlife projects at the Würzburg study site, and critically revised the manuscript. JG coordinated the Würzburg study site, contributed to the management and organization of the ESCAlife projects, and critically revised the manuscript. LP heads the Göttingen study site of ESCAschool, was involved in the implementation of the ESCAlife projects, and critically revised the manuscript. HU coordinated ESCAschool at the Göttingen study site, contributes to the management and organization of the study, was involved in patient recruitment and data acquisition, and critically revised the manuscript. TR heads the Tübingen study site of ESCAschool, contributed to the implementation of the ESCAschool study, and critically revised the manuscript. UD coordinates the Tübingen study site of ESCAschool, contributes to the management and organization of the study, was involved in patient recruitment and data acquisition, and critically revised the manuscript. All authors gave final approval of the last version of the manuscript and agreed to be accountable for all aspects of the work in ensuring that questions related to the accuracy or integrity of any part of the work are appropriately investigated and resolved.

## Conflict of Interest

A-KTh, AG-D, and MD were involved in the development of the DISYPS-ILF and will receive royalties after publication of this instrument from the publisher Hogrefe. AG-D and MD are AKiP supervisors and lecturers and received income as heads of the School for Child and Adolescent Behavior Therapy at the University of Cologne and royalties from treatment manuals, books and psychological tests published by Guilford, Hogrefe, Enke, Beltz, and Huber. MD received consulting income and research support from Lilly, Medice, Shire, Janssen Cilag, Novartis, and Vifor. AG-D is head of the AKiP Research and Evaluation department. TB served in an advisory or consultancy role for Lundbeck, Medice, Neurim Pharmaceuticals, Oberberg GmbH, Shire, and Infectopharm; and received conference support or speaker’s fees from Lilly, Medice, and Shire; and received royalties from Hogrefe, Kohlhammer, CIP Medien, and Oxford University Press. DB served as an unpaid scientific advisor for an EU-funded neurofeedback trial unrelated to the present work. KB has been involved in research/clinical trials with Eli Lilly (≤2011) and Shire (2010), was on the Advisory Board of Eli Lilly/Germany (≤2014), a member of the Scientific Committee of Shire (≤2012) and was paid for public speaking by Eli Lilly (<2011) and Shire. These activities do not bias the objectivity of this manuscript (in her opinion), but are mentioned for the sake of completeness. MHo served in an advisory role for Shire and Medice and received conference attendance support or was paid for public speaking by Medice, Shire and Neuroconn. He receives research support from the German Research Foundation and the German Ministry of Education and Research. He receives royalties as editor in chief of the German Journal for Child and Adolescent Psychiatry and for text books from Hogrefe. MHu served as a member of the advisory boards of Eli Lilly and Co., Engelhardt Arzneimittel, Janssen-Cilag, Medice, Novartis, Shire, and Steiner Arzneimittel within the past 5 years; served as a consultant to Engelhardt Arzneimittel, Medice, and Steiner Arzneimittel; received honoraria from Eli Lilly and Co., Engelhardt Arzneimittel, Janssen-Cilag, Medice, Novartis, and Shire; and received unrestricted grants for investigator-initiated trials from Eli Lilly and Co., Medice, Engelhardt Arzneimittel, and Steiner Arzneimittel. LP served in an advisory or consultancy role for Shire, Roche and Infectopharm; and received speaker’s fees from Shire and royalties from Hogrefe, Kohlhammer, and Schattauer. HU served in an advisory or consultancy role for Medice; and received speaker’s fees from Shire and Medice.

The remaining authors declare that the research was conducted in the absence of any commercial or financial relationships that could be construed as a potential conflict of interest.
