# Supplementary material for: Toward a Dimensional Assessment of Externalizing Disorders in Children: Reliability and Validity of a Semi-Structured Parent Interview
Source: Front Psychol. 2020 Jul 24;11:1840. doi: 10.3389/fpsyg.2020.01840 (PMC7396521; doi:10.3389/fpsyg.2020.01840)
Supplement: Supplementary file 1 [file Table_1.docx]

Supplementary Material

**Supplemental Table 1:** Scale composition and interrater reliability of the ILF-EXTERNAL on the single item level

| **No.** | **Item** | **ICC(1,1)** | **95% CI** | **ICC(1,3)** | **95% CI** | **Pairwise percent agreement** |
| --- | --- | --- | --- | --- | --- | --- |
| ADHD Symptoms (A01-A09; B01-B09) | | .91 | .87 - .95 | .97 | .95 - .98 | 88.1 |
| - Inattention (A01-A09) | | .83 | .74 - .90 | .94 | .89 - .96 | 85.2 |
| A01 | Careless | .73 | .60 - .83 | .89 | .82 - .94 | 76.3 |
| A02 | Sustaining attention | .48 | .30 - .65 | .73 | .57 - .85 | 68.9 |
| A03 | Does not listen | .80 | .70 - .88 | .92 | .87 - .96 | 71.1 |
| A04 | Does not finish work | .69 | .55 - .80 | .87 | .78 - .92 | 72.6 |
| A05 | Organizational skills | .71 | .58 - .82 | .88 | .81 - .93 | 68.9 |
| A06 | Concentration | .74 | .61 - .83 | .89 | .83 - .94 | 68.2 |
| A07 | Loses things | .84 | .76 - .90 | .94 | .90 - .97 | 69.6 |
| A08 | Easily distracted | .64 | .49 - .77 | .84 | .74 - .91 | 73.3 |
| A09 | Forgetful | .76 | .64 - .85 | .90 | .84 - .94 | 62.2 |
| - Hyperactivity-Impulsivity (B01-B09) | | .95 | .91 - .97 | .98 | .97 - .99 | 82.2 |
| B01 | Fidgets | .88 | .82 - .93 | .96 | .93 - .98 | 82.2 |
| B02 | Leaves seat | .84 | .75 - .90 | .94 | .90 - .97 | 74.1 |
| B03 | Runs / climbs | .79 | .68 - .87 | .92 | .86 - .95 | 69.6 |
| B04 | Playing quietly | .77 | .66 - .86 | .91 | .85 - .95 | 65.9 |
| B05 | Driven / on the go | .90 | .85 - .94 | .96 | .74 - .98 | 75.6 |
| B06 | Blurts out answers | .86 | .79 - .92 | .95 | .92 - .97 | 70.4 |
| B07 | Awaiting turn | .87 | .80 - .92 | .95 | .92 - .97 | 71.9 |
| B08 | Interrupts / intrudes | .80 | .70 - .88 | .93 | .88 - .86 | 73.3 |
| B09 | Talks excessively | .86 | .78 - .91 | .95 | .91 - .97 | 77.8 |
| ADHD Functional Impairment (F01-F05) (*n* = 39) | | .89 | .82 - .94 | .96 | .93 - .98 | 80.7 |
| F01 | Psychological strain (*n* = 43) | .85 | .77 - .91 | .95 | .91 - .97 | 76.7 |
| F02 | **﻿**Interferes with home life and family members (*n* = 42) | .77 | .65 - .86 | .91 | .85 - .95 | 74.8 |
| F03 | ﻿Interferes with adult interaction (*n* = 42) | .82 | .72 - .89 | .93 | .88 - .96 | 75.6 |
| F04 | ﻿Interferes with child interactions; ﻿interferes with ﻿recreational activities (*n* = 42) | .84 | .75 - .90 | .94 | .90 | 72.4 |
| F05 | Interferes with educational activities (*n* = 39) | .77 | .66 - .87 | .91 | .81 - .95 | 71.5 |
| ODD/CD Symptoms - short version (A01-A08; B01-B05) | | .94 | .90 - .96 | .98 | .97 - .99 | 91.1 |
| - ODD Symptoms (A01-A08) | | .94 | .90 - .96 | .98 | .96 - .99 | 83.7 |
| A01 | Loses temper^1^ | .84 | .76 - .90 | .94 | .90 - .97 | 70.4 |
| A02 | Touchy / easily annoyed^1^ | .89 | .83 - .93 | .96 | .94 - .98 | 80.0 |
| A03 | Angry / resentful^1^ | .87 | .80 - .92 | .95 | .93 - .97 | 78.5 |
| A04 | Argues with adults | .83 | .74 - .90 | .94 | .90 - .96 | 76.3 |
| A05 | Complies with requests | .80 | .70 - .88 | .92 | .88 - .96 | 69.6 |
| A06 | Annoys | .89 | .83 - .93 | .96 | .94 - .98 | 78.5 |
| A07 | Blames others | .88 | .81 - .92 | .95 | .93 - .98 | 78.5 |
| A08 | Spiteful / vindictive | .90 | .85 - .94 | .97 | .94 - .98 | 91.1 |
| - CD Symptoms - short version (B01-B05) | | .90 | .85 - .94 | .97 | .94 - .98 | 88.2 |
| B01 | Physical fights | .88 | .81 - .93 | .96 | .93 - .98 | 88.9 |
| B02 | Bullies, threatens, or intimidates (*n* = 44) | .85 | .77 - .91 | .95 | .91 - .97 | 79.5 |
| B03 | Cruel to animals | .90 | .85 - .94 | .97 | .94 - .98 | 94.1 |
| B04 | Lies | .77 | .65 - .85 | .91 | .85 - .95 | 65.9 |
| B05 | Steals without confrontation | .78 | .67 - .86 | .91 | .86 - .95 | 86.7 |
| B06 | Uses weapon in fight^2^ |  | - | - | - | - |
| B07 | Cruel to people^2^ | - | - | - | - | - |
| B08 | Steals with confrontation^2^ | - | - | - | - | - |
| B09 | Sexual assault^2^ | - | - | - | - | - |
| B10 | Fire setting^2^ | - | - | - | - | - |
| B11 | Vandalism^2^ | - | - | - | - | - |
| B12 | Breaking in^2^ | - | - | - | - | - |
| B13 | Stays out at night^2^ | - | - | - | - | - |
| B14 | Runs away from home overnight^2^ | - | - | - | - | - |
| B15 | Truancy^2^ | - | - | - | - | - |
| Disruptive Mood Dysregulation (D01-D02; A01-A03) | | .90 | .85 - .94 | .97 | .94 - .98 | 83.7 |
| D01 | Recurrent temper outbursts | .74 | .61 - .83 | .89 | .83 - .94 | 69.6 |
| D02 | Persistently irritable or angry mood | .70 | .57 - .81 | .88 | .80 - .93 | 65.2 |
| Limited Prosocial Emotions (C01a-C04d) (*n* = 41) | | .93 | .89 - .96 | .98 | .96 - .99 | 86.7 |
| C01a | Lack of remorse / guilt (*n* = 44) | .92 | .88 - .96 | .97 | .96 - .98 | 92.4 |
| C01b | Lack of concern (*n* = 44) | .89 | .82 - .93 | .96 | .93 - .98 | 80.3 |
| C02a | Cold and uncaring (*n* = 44) | .89 | .83 - .94 | .96 | .94 - .98 | 85.6 |
| C02b | Self-serving (*n* = 43) | .92 | .88 - .96 | .97 | .96 - .99 | 87.8 |
| C03a | Indifferent of poor performance (*n* = 42) | .86 | .78 - .92 | .95 | .91 - .97 | 84.1 |
| C03b | Avoids effort (*n* = 42) | .85 | .76 - .91 | .94 | .90 - .97 | 76.2 |
| C03c | Blames others for poor performance (*n* = 42) | .87 | .80 - .93 | .95 | .92 - .97 | 76.2 |
| C04a | Shallow / deficient affect (*n* = 42) | .69 | .56 - .81 | .87 | .79 - .93 | 78.6 |
| C04b | Turns emotions ‘on’ or ‘off’ quickly (*n* = 40) | .81 | .71 - .89 | .93 | .88 - .96 | 80.8 |
| C04c | Manipulates (*n* = 41) | .83 | .73 - .90 | .94 | .89 - .96 | 83.7 |
| C04d | Inconsistent affect (*n* = 41) | .87 | .80 - .93 | .95 | .92 - .97 | 93.5 |
| ODD/CD Functional Impairment (F01-F05) (*n* = 31) | | .92 | .86 - .96 | .97 | .95 - .99 | 85.2 |
| F01 | Psychological strain (*n* = 33) | .79 | .66 - .88 | .92 | .85 - .96 | 72.3 |
| F02 | ﻿Interferes with home life and family members (*n* = 32) | .80 | .67 - .89 | .92 | .86 - .96 | 75.2 |
| F03 | ﻿Interferes with adult interaction (*n* = 32) | .81 | .69 - .89 | .93 | .87 - .96 | 68.0 |
| F04 | ﻿Interferes with child interactions; ﻿interferes with ﻿recreational activities (*n* = 31) | .89 | .81 - .94 | .96 | .93 - .98 | 80.8 |
| F05 | Interferes with educational activities (*n* = 31) | .88 | .80 - .94 | .96 | .92 - .98 | 81.9 |

*Note.* ADHD = attention-deficit/hyperactivity disorder; CD = conduct disorder; CI = confidence interval; ICC = Intraclass correlation; ICC(1,1) = one-way random-effects, absolute agreement model for single rater/measurements; ICC(1,3) = one-way random-effects, absolute agreement model ﻿based on a mean-rating; ODD = oppositional defiant disorder; n = 45 (if not otherwise specified).
^1^Items also included in the Disruptive Mood Dysregulation scale.
^2^The items B06 to B15 assessing aggressive and antisocial symptoms from the age of 11 were excluded from further analyses due to an obvious floor effect.

**Supplemental Table 2:** Interrater reliability of the ILF-EXTERNAL scales between two independent raters

| **Scale** | **ICC(2,1)** | **95% CI** | **ICC(2,2)** | **95% CI** | **Pairwise percent agreement** | **n** |
| --- | --- | --- | --- | --- | --- | --- |
| ADHD Symptoms | .95 | .83 - .98 | .97 | .91 - .99 | 86.7 | 45 |
| - Inattention | .84 | .71 - .91 | .91 | .83 - .95 | 82.2 | 45 |
| - Hyperactivity-Impulsivity | .96 | .91 - .98 | .98 | .95 - .99 | 75.6 | 45 |
| ADHD Functional Impairment | .90 | .82 - .95 | .95 | .90 - .97 | 75.6 | 39 |
| ODD/CD Symptoms - short version | .96 | .92 - .98 | .98 | .96 - .99 | 86.7 | 45 |
| - ODD Symptoms | .95 | .90 - .97 | .97 | .95 - .99 | 84.4 | 45 |
| - CD Symptoms - short version | .91 | .84 - .95 | .95 | .91 - .97 | 86.7 | 44 |
| Disruptive Mood Dysregulation | .91 | .85 - .95 | .96 | .92 - .98 | 80.0 | 45 |
| Limited Prosocial Emotions | .97 | .94 - .98 | .98 | .97 - .99 | 82.2 | 41 |
| ODD/CD Functional Impairment | .92 | .84 - .96 | .96 | .91 - .98 | 77.8 | 31 |

*Note.* ADHD = attention-deficit/hyperactivity disorder; CD = conduct disorder; CI = confidence interval; ICC = Intraclass correlation; ICC(2,1) = two-way random-effects, absolute agreement model for single rater/measurements; ICC(2,2) = two-way random-effects, absolute agreement model based on a mean-rating; ODD = oppositional defiant disorder.
